# A broader neutralizing antibody against all the current VOCs and VOIs targets unique epitope of SARS-CoV-2 RBD

**DOI:** 10.1038/s41421-022-00443-w

**Published:** 2022-08-17

**Authors:** Shuo Liu, Zijing Jia, Jianhui Nie, Ziteng Liang, Jingshu Xie, Lei Wang, Li Zhang, Xiangxi Wang, Youchun Wang, Weijin Huang

**Affiliations:** 1 Division of HIV/AIDS and Sex-transmitted Virus Vaccines, Institute for Biological Product Control, National Institutes for Food and Drug Control (NIFDC), Beijing, China; 2 CAS Key Laboratory of Infection and Immunity, National Laboratory of Macromolecules, Institute of Biophysics, Chinese Academy of Sciences, Beijing, China; 3 Graduate School of Chinese Academy of Medical Sciences & Peking Union Medical College, Beijing, China; 4 Beijing Biocytogen Co., Ltd, Beijing, China

**Keywords:** Electron microscopy, Autoimmunity

Dear Editor,

As of January 31, 2022, the COVID-19 pandemic has caused more than 375 million infections and over 5.6 million deaths (https://covid19.who.int), which has posed an unprecedented impact on the global economy and people’s lives. With the continuous evolution of SARS-CoV-2 giving rise to new variants, the pandemic has not slowed down at all. The variations result in a change of the infectivity and antigenicity of the virus, which leads to the continuous replacement of the epidemic strain or the co-circulation of multiple variants. In particular, some mutations enable the virus to escape immune response induced by the original strain, resulting in a decline of the protective effect of the existing vaccines and reinfection of those infected with the original strain^1–3^. This poses a greater challenge to control the pandepidemic.

Neutralizing monoclonal antibodies (mAbs) against SARS-CoV-2 played an important role in the treatment of COVID-19, especially in early infection and pre-exposure prevention^4^. As of now, five therapeutic mAbs have been approved for the treatment of COVID-19 by the FDA and EMA. All these mAbs target the RBD region of S protein which is crucial for establishing viral infection^4^. However, the neutralization efficiency of many mAbs has been greatly reduced against the newly emerged variants^5^. Mutations in these variants may be located in the binding epitopes of mAbs, or may cause conformational changes in S proteins, resulting in changes in the accessibility of binding epitopes, affecting the neutralization of the virus.

Combination of monoclonal antibodies, the so-called cocktail therapy, has been shown to be effective, to some extent, in offsetting the reduction in neutralization efficiency caused by mutations. Another widely practiced strategy to counter mutations is to screen for conserved antibody-binding epitopes that have not been targeted as yet. In a previous study, we demonstrated a cocktail of antibodies formulated by combining antibodies targeting different epitopes in improving the range and neutralization potency of antibody therapy. At that time, the study only focused on Alpha and Beta variants, as well as some sites with higher mutation frequency^6^. In this work, we tested a set of 10 neutralizing mAbs again against all the current variants of concern (VOCs) and variants of interest (VOIs) using pseudotyped viruses. We found that SARS-CoV-2 variants, especially the Omicron variant, could escape neutralization by 9 of the 10 mAbs derived from humanized mice to varying degrees (Fig. 1a; Supplementary Fig. S1). Incidentally, these included antibodies that we used previously for designing a widely effective cocktail (10D12 + 7B8 + 9G11) (Supplementary Table S1). Therefore, this cocktail is likely to show reduced efficacy against Omicron. The neutralizing activity of three commercially available antibodies, LY-CoV555, REGN10987, and REGN10933, against Omicron also decreased significantly. Surprisingly, one mAb, 9A8, among the ten tested, showed high neutralizing activity against all VOCs and VOIs, with the IC_50_ being less than 0.1 μg/mL (Fig. 1a; Supplementary Fig. S1 and Table S2). In fact, there was no decrease in sensitivity to neutralization between different variants and the wild-type (WT) strain. To verify the broad-spectrum neutralization activity of mAb 9A8, we tested all the mAbs again against live WT as well as Beta, Delta, and Omicron variants. As observed in the pseudovirus-based assays, mAb 9A8 showed the best neutralization activity against all the live viruses tested. The IC_50_ value of neutralization for 9A8 against Omicron was 0.49 μg/mL, while that for the other 9 mAbs was greater than 125 μg/mL, suggesting a loss in their ability to neutralize Omicron (Fig. 1b; Supplementary Table S3). These results indicate that the epitope targeted by 9A8 is probably unique, which permitted it to efficiently neutralize the VOCs and VOIs.

**Fig. 1:**
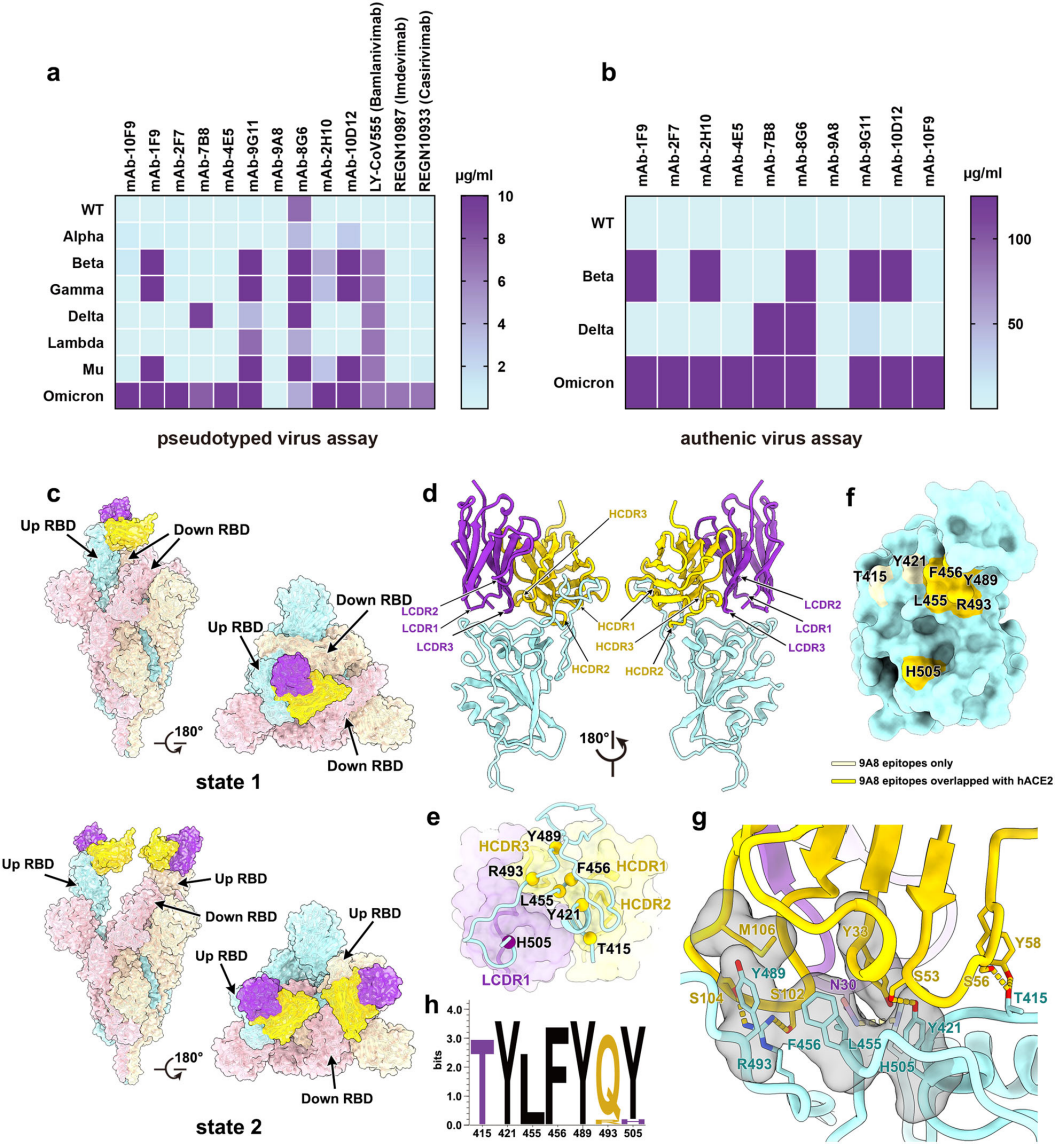
Neutralization activities of monoclonal antibodies against circulating variants and the structure of Omicron S in complex with 9A8. **a** Susceptibility of VOCs and VOIs to neutralization by mAbs tested in the pseudotyped virus neutralization assay^11^. **b** Three VOCs’ susceptibility to the mAbs in authentic virus assay^12^. **c** Side view and top view of surface representations of the structures of SARS-CoV-2 Omicron S trimer in complex with Fab 9A8 (state 1: one up RBD; state 2: two up RBDs) with different colors for each S monomer (pink, light yellow, cyan) and Fab 9A8 (light chain: purple; heavy chain: yellow). **d** Side views of the binding interface to show the binding mode of 9A8. **e** Cartoon representation of the interacting residues in RBD and CDRs in 9A8. The residues (T415, Y421, L455, F456, Y489, R493, H505) comprising the epitope are shown as spheres and labeled. The 9A8 Fab is presented as surface with 30% transparency and CDRs involved in the interaction with RBD are highlighted. **f** Surface representations of the SARS-CoV-2 Omicron RBD. Residues colored in yellow (pale and bright yellow) are the residues recognized by 9A8. Among these, residues overlapping with binding sites for ACE2 are colored in bright yellow. **g** Details of the interactions between 9A8 and SARS-CoV-2 Omicron RBD. Residues involved in the formation of hydrogen bonds are shown as sticks and labeled. Hydrophobic patches are shown in gray surface representation. Hydrogen bonds are depicted as yellow dots. The color scheme is the same as in **c**. **h** Sequence conserved analysis of 9A8 epitope. The logo plots represent the conservation of the epitope of 9A8 from 18 SARS-CoV-2, including WT, VOCs (Alpha, Beta, Gamma, Delta, Omicron), VOIs (Lambda, Mu) and other variants (Delta plus, Eta, Lota, Kappa, Theta, Iota, B.1.1.318, B.1.620, C.1.2 and C.363).

To delineate the structural basis for 9A8-mediated broad neutralization, we characterized cryo-EM structures of a prefusion stabilized Omicron S trimer in complex with the 9A8 Fab fragment. Similar to previous studies, 3D classification revealed that the complex adopts two distinct conformational states, corresponding to one up RBD and two down RBDs (state 1) and two up RBDs and one down RBD (state 2) (Fig. 1c; Supplementary Fig. S2). We obtained asymmetric reconstructions at 3.4 Å and 3.6 Å resolution of the Omicron–9A8 complex in states 1 and 2, respectively (Supplementary Fig. S3; Supplementary Table S4). To improve the local resolution, we performed local refinement by using an optimized “block-based” reconstruction approach, enabling reliable analysis of the interaction interface (Supplementary Fig. S4).

9A8 binds to the apical tip of RBD, partially overlapping with the receptor-binding motif (RBM) (Fig. 1d, e). The distal tip of the RBM inserts into the cavity constructed by four complementarity-determining regions (HCDR1–3 and LCDR1), involving extensively hydrophobic interactions (Fig. 1e, f). The 9A8 epitope includes only 7 residues, forming a relatively small patch, which largely overlaps with the binding region targeted by ACE2 (Fig. 1e, f). Tight binding is achieved by a network of hydrophobic interactions formed by Y421, L455, F456 and Y489 from Omicron RBD with Y33, Y58, G101 and M106 from the heavy chain (Fig. 1g; Supplementary Table S5). Additionally, six hydrogen bonds formed by T415, Y421, R493 and H505 from RBD and S56, Y58, S53, S102, S104 and N30 from CDRs further facilitate the interactions (Fig. 1g). Of note, 7 residues comprising the epitope are mostly conserved with two single-site mutations (Q493R and Y505H) among all circulating SARS-CoV-2 variants, informing its neutralizing breadth for SARS-CoV-2 variants (Fig. 1h).

According to the previously reported classification based on binding epitopes^7–9^, mAb 9A8 belongs to class II antibodies (Supplementary Fig. S5). These antibodies are usually characterized by high binding affinities and neutralizing activities^7,9^. Among the 7 residues of RBD involved in binding 9A8, 5 are immunogenic hot binding residues^10^, including L455, F456, Y489, R493, and H505, which indicates a low probability of mutagenesis of the above five loci in variants. Except for the substitutions of Q493R and Y505H observed in Omicron or a similar mutation, Q493K, previously detected through in vitro resistance mapping efforts or in immunocompromised hosts, the other 5 loci do not appear to be mutated in any of the currently known VOCs or VOIs. More importantly, Q493R and Y505H do not affect the neutralization activity of 9A8, revealed by its comparable neutralizing activities against WT and Omicron strain. Thus, the reason underlying the highly efficient neutralization activity of mAb 9A8 against all known variants of SARS-CoV-2 lies in the nature of the epitope targeted, which is importantly conserved for most part.

The nature of the epitope has a profound impact on the potency and neutralization breadth of an antibody. The epitope targeted by mAb 9A8 seems mostly conserved, conferring two highly sought characteristics in antibodies for treating COVID-19: (i) high neutralization efficiency and (ii) broad spectrum of neutralization capable of neutralizing all known variants. Moreover, the molecular features of the 9A8 epitope unveiled in this study pose interesting targets for structure-based rational vaccine design. We have not only discovered a highly efficient broad-spectrum SARS-CoV-2 neutralizing mAb, but the structure of the mAb bound with Omicron S described in this study is instructive for structure-based vaccine design for ending the pandemic.

## Supplementary information

Supplementary Information

## Data Availability

The atomic coordinates of the Omicron S-trimer in complex with 9A8 (state 1 and 2) and the focused refined binding interface have been submitted to the Protein Data Bank with accession numbers: 7XXX, 7YYY and 7ZZZ, respectively. Also, their corresponding cryo-EM maps have been deposited in the Electron Microscopy Data Bank under accession codes: EMD-XXXX, EMD-YYYY and EMD-ZZZZ, respectively. Besides, all the other materials and data involved in this study will be available upon request.

## Materials availability

Correspondence and requests for materials should be addressed to Y.W., X.W., or W.H.
