# Author Correction: A broader neutralizing antibody against all the current VOCs and VOIs targets unique epitope of SARS-CoV-2 RBD

**DOI:** 10.1038/s41421-023-00619-y

**Published:** 2023-12-08

**Authors:** Shuo Liu, Zijing Jia, Jianhui Nie, Ziteng Liang, Jingshu Xie, Lei Wang, Li Zhang, Xiangxi Wang, Youchun Wang, Weijin Huang

**Affiliations:** 1 Division of HIV/AIDS and Sex-transmitted Virus Vaccines, Institute for Biological Product Control, National Institutes for Food and Drug Control (NIFDC), Beijing, China; 2 CAS Key Laboratory of Infection and Immunity, National Laboratory of Macromolecules, Institute of Biophysics, Chinese Academy of Sciences, Beijing, China; 3 Graduate School of Chinese Academy of Medical Sciences & Peking Union Medical College, Beijing, China; 4 Beijing Biocytogen Co., Ltd, Beijing, China

**Keywords:** Electron microscopy, Autoimmunity

Correction to: *Cell Discovery*

10.1038/s41421-022-00443-w published online 17 August 2022

In the original publication of the article, the authors have found several mistakes in Data availability. The modified Data availability is described below:

**Data availability**

The atomic coordinates of the Omicron S-trimer in complex with 9A8 (state I and II) and the focused refined binding interface have been submitted to the Protein Data Bank with accession numbers: 7WT7, 7WT8 and 7WT9, respectively. Also, their corresponding cryo-EM maps have been deposited in the Electron Microscopy Data Bank under accession codes: EMD-32770, EMD-32771 and EMD-32772, respectively. Besides, all the other materials and data involved in this study will be available on request.

