# Supplementary material for: A broader neutralizing antibody against all the current VOCs and VOIs targets unique epitope of SARS-CoV-2 RBD
Source: Cell Discov. 2022 Aug 17;8:81. doi: 10.1038/s41421-022-00443-w (PMC9385086; doi:10.1038/s41421-022-00443-w)
Supplement: Supplementary file 1 — Supplementary Information [file 41421_2022_443_MOESM1_ESM.pdf]

Supplementary methods

Pseudotyped virus neutralization assay

The pseudotyped virus neutralization was performed as described previously[1]. Briefly, the monoclonal antibodies were serially diluted and added into 96-well cell culture plates in 100 µl. Then, 50 µl SARS-CoV-2 pseudotyped viruses were added into the plates with a concentration of 1200 $TCID_{50}$/ml, followed by incubation for 1 h at 37°C. Next, 100 µl Huh-7 cells were added into the plate with a concentration of 2-3×10^5 cells/ml, followed by incubation at 37°C with 5% CO2. After 24 h of incubation, chemiluminescence detection was conducted using a luminometer (PerkinElmer, Ensight), and the EC_{50} of antibody was calculated by Reed-Muench method[11].

Live virus neutralization test

The live virus neutralization was performed as described below. Briefly, 50 µl serially diluted monoclonal antibodies were added into 96-well cell culture plates. Then, 50 µl SARS-CoV-2 authentic viruses were added into the plates with a concentration of 2000 $CCID_{50}$/ml, followed by incubation for 1-1.5 h at 36.5±1°C. Next, 100 µl Vero cells were added into the plate with a concentration of 1-2×10^5 cells/ml, followed by incubation at 36.5±1°C with 5% CO2. After 5 d of incubation, the cytopathic effect (CPE) of each well was observed under microscopes by three different individuals and the EC_{50} were calculated by the method of Reed-Muench[12].

Cryo-EM sample preparation and data collection

The purified Omicron S trimer was mixed with 9A8 with a molar ratio of 1:1.2 for 10 s ice incubation. Then 3 µl of the mixture was deposited onto the glow-discharged gold grid (C-flat, 300-mesh, 1.2/1.3, Protochips In). After blotting for 6 s in 100% relative humidity immediately plunge into the liquid ethane using Vitrobot (FEI). Cryo-EM data were collected at 300kV using an FEI Titan Krios microscope (FEI). Movies (32 frames, each 0.2 s, total dose of 60 e^- $Å^{-2}$) were recorded using a K3 Summit direct detector with a defocus range between 1.5-2.7 µm. Automated single particle data acquisition was carried out by SerialEM, with a calibrated magnification of 22,500 yielding a final pixel size of 1.07 Å.

Cryo-EM data processing

A total of 5,061 Micrographs was collected and subjected to beam-induced motion correction using MotionCorr2 in Relion3.0 package. Then the defocus value of each micrograph was estimated by Gctf. 2,580,638 particles were autopicked and extracted. All the particles were applied to 2D classification by cryoSPARC and 3D classification by Relion3.0. After that, 325,941 and 174,759 particles were selected and processed by non-uniform auto-refinement and postprocessing in cryoSPARC respectively to generate the final cryo-EM density. To improve the resolution of the binding interface of SARS-CoV-2 Omicron RBD and 9A8, Local refinement was performed. The resolution of each structure was determined on the basis of the gold-standard Fourier shell correlation (threshold = 0.143) and evaluated by ResMap.

Model fitting and refinement

The atomic models of the complex were built first by fitting the chain of the apo SARS-CoV-2 S trimer (PDB: 6VYB) and Fabs (PDB: 7BZ5 and 2Q20) into the final cryo-EM density by Chimera. Then Manually adjust and correct according to the protein sequences and density in Coot, while use real space refinement in Phenix.

Supplementary Figures

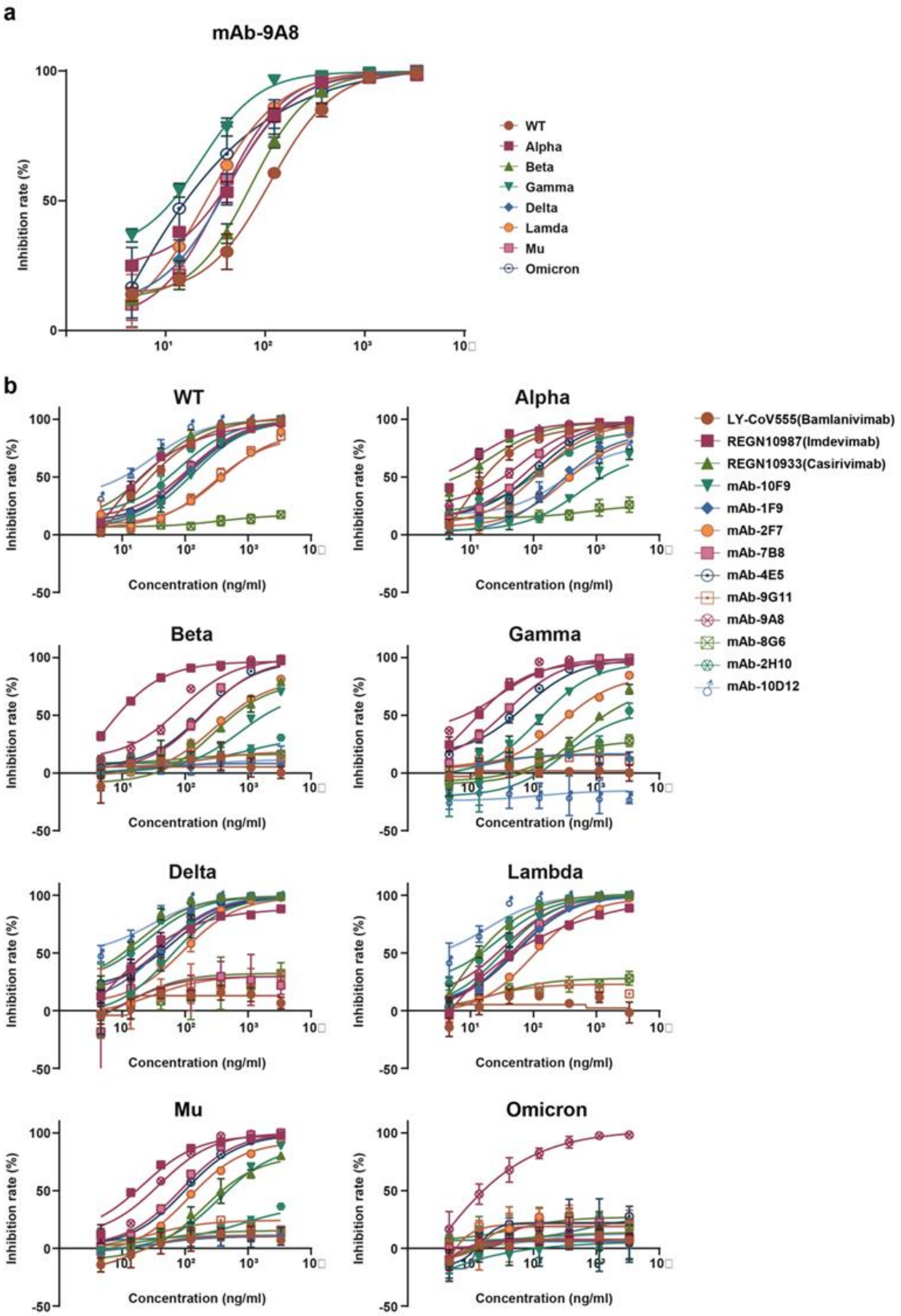

Fig. S1 Neutralization curves of the mAbs against pseudotyped viruses.

a Neutralization curves for 9A8 on pseudotyped viruses with the S protein of wild-type or variants of concern or interest (Alpha, Beta, Gamma, Delta, Lambda, Mu and Omicron).

b Neutralization curves of the representative mAbs against the VOCs and VOIs.

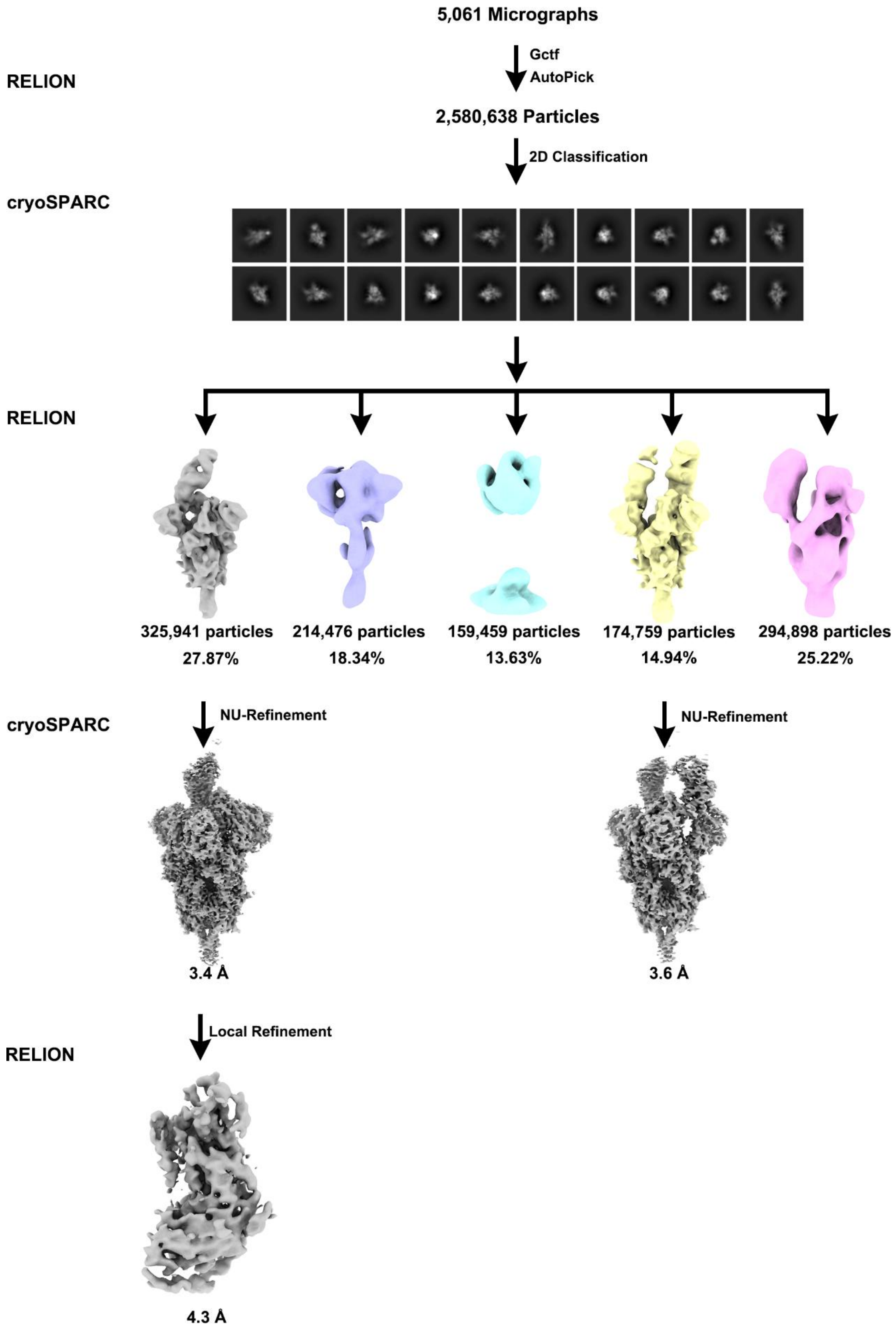

Fig. S2 Flowcharts for SARS-CoV-2 Omicron S trimer in complex with 9A8

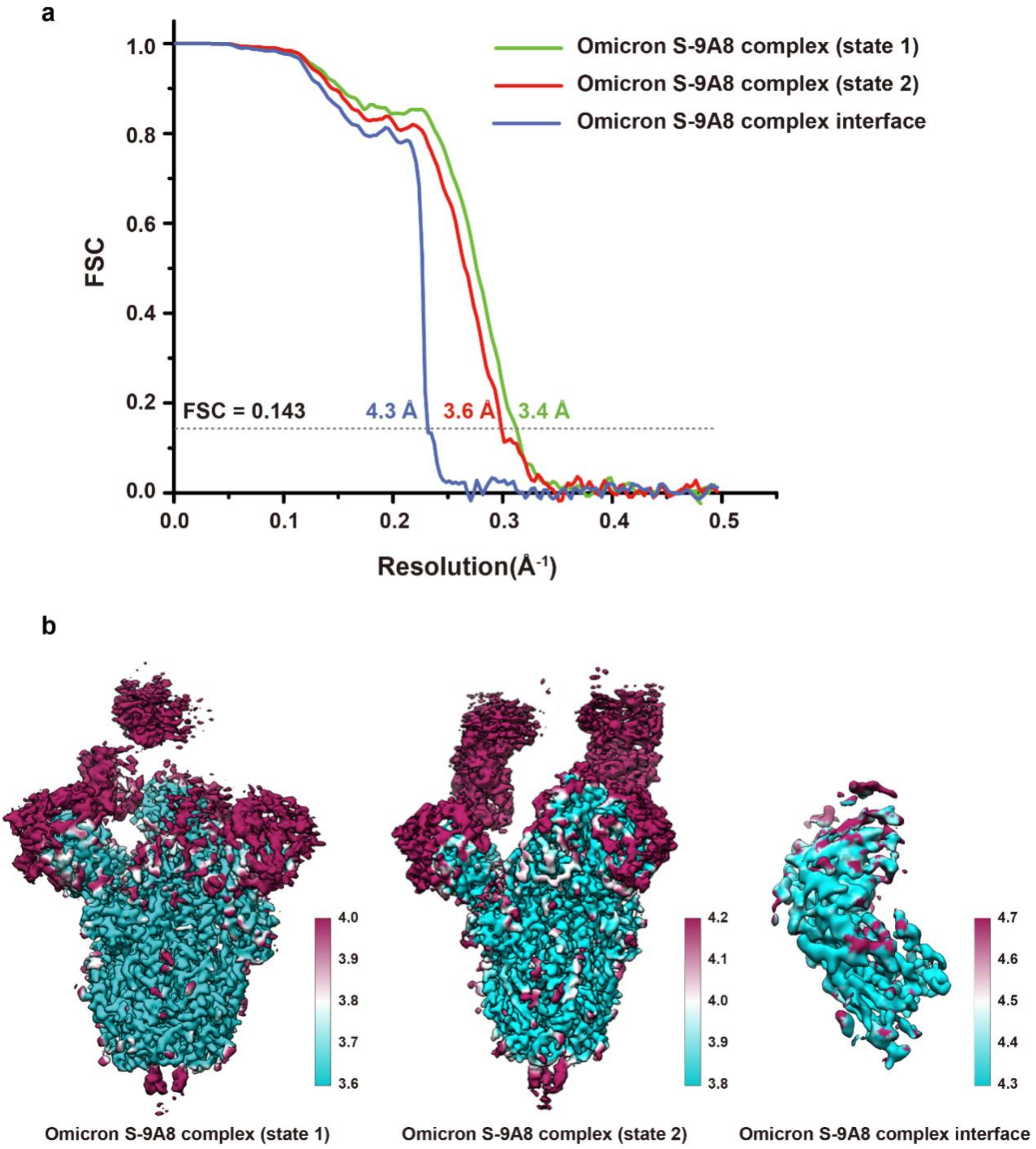

Fig. S3 Resolution estimation of the EM maps.

a The gold-standard FSC curves of overall maps of Omicron S trimer in complex with Fab 9A8 and local map of interface. **b** Local resolution assessments of cryo-EM maps using ResMap are shown.

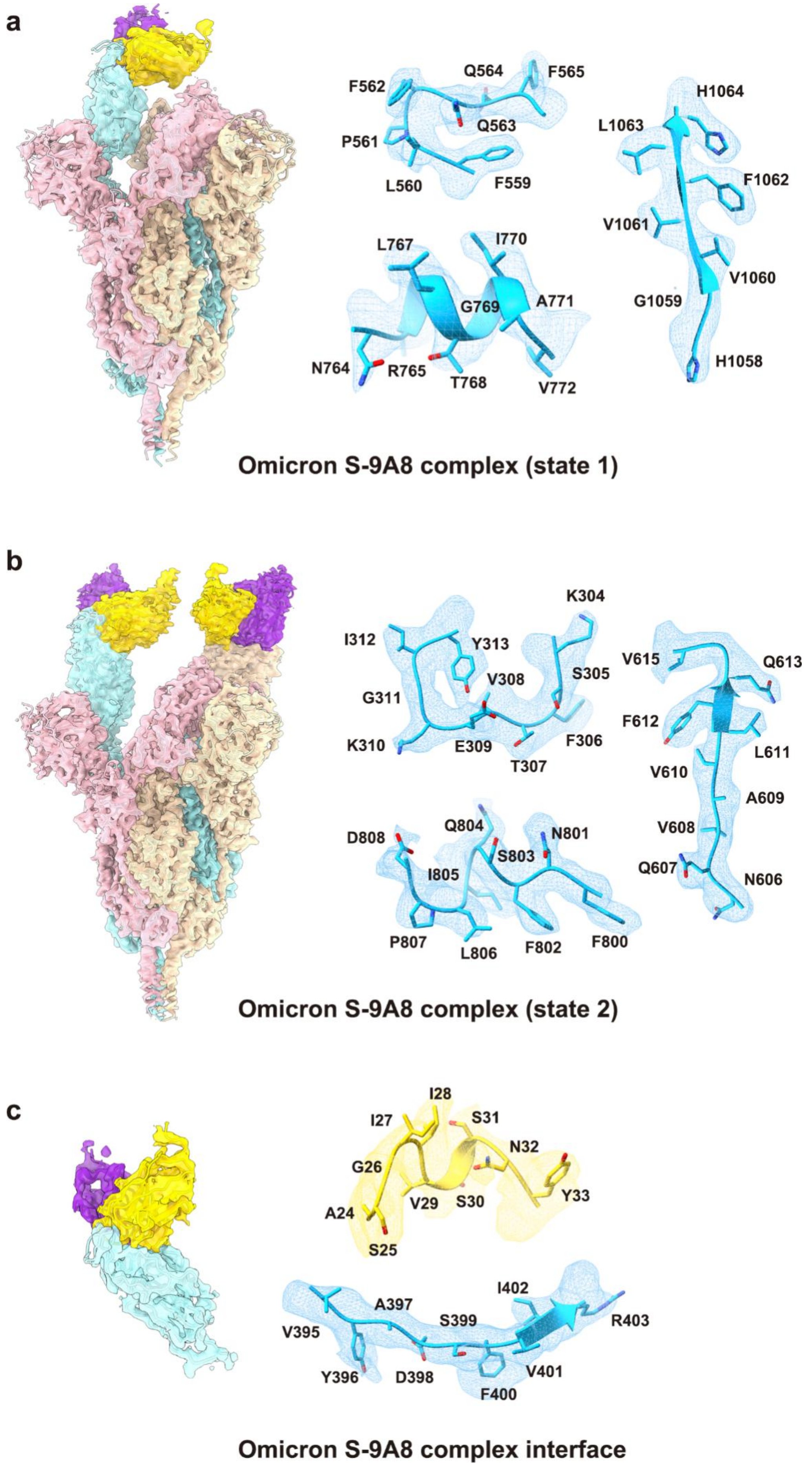

a
F562
Q564
F565
H1064
Q563
L1063
P561
L560
F559
F1062
L767
I770
V1061
G769
A771
V1060
G1059
N764
R765
T768
V772
H1058
Omicron S-9A8 complex (state 1)
b
K304
I312
Y313
V308
V615
Q613
S305
G311
F612
K310
E309
F306
T307
L611
V610
A609
Q804
N801
D808
V608
S803
I805
Q607
N606
P807
L806
F802
F800
Omicron S-9A8 complex (state 2)
c
I28
I27
S31
N32
G26
V29
S30
Y33
A24
S25
I402
A397
S399
R403
V395
Y396
D398
F400
V401
Omicron S-9A8 complex interface

Fig. S4 Density maps and atomics models.

Cryo-EM density maps of Omicron S trimer in complex with 9A8 and their interfaces are shown. Color scheme is the same as in **Fig. 1**. Residues are shown as sticks with oxygen colored in red, nitrogen colored in blue and sulfurs colored in yellow.

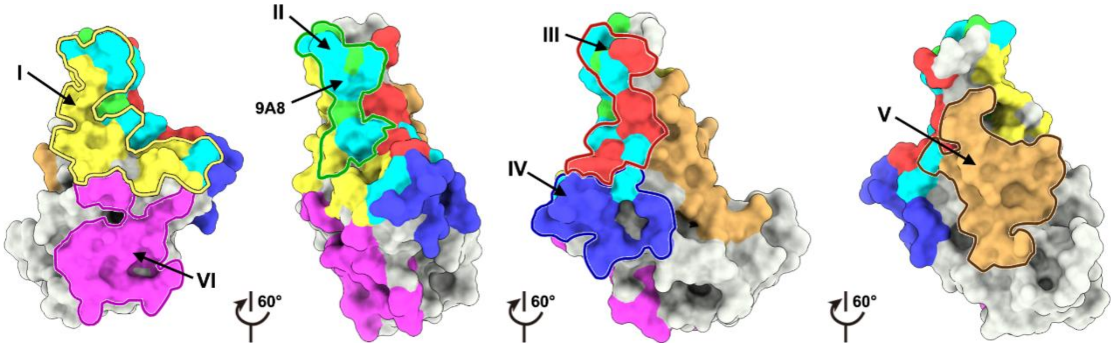

Fig. S5 Structural landscapes of the six classes of RBD mAbs

Surface representative model of RBD colored by six classes of antibodies. Residues colored by yellow, magenta, green, red, blue and brown represent epitopes of each class of antibody. Residues colored by cyan represent epitopes identified by more than two classes of antibodies. Structural landscapes of the six classes of RBD mAbs are circled by lines colored by yellow, magenta, green, red, blue and brown. 9A8 belongs to class II.

Table S1. Neutralizing titers of mAb-9A8 and mAb-10D12+7B8+9G11 in the WT,BA.1,BA.2 pseudotyped virus neutralization assay

	mAb-9A8	mAb-10D12+7B8+9G11
WT（IC50μg/ml）	0.07	0.06
BA.1（IC50μg/ml）	0.02	>3.33
BA.2（IC50μg/ml）	0.07	>3.33

Table S2. Neutralizing titers of the mAbs in the pseudotyped virus neutralization assay

	mAb-10F9	mAb-1F9	mAb-2F7	mAb-7B8	mAb-4E5	mAb-9G11	mAb-9A8	mAb-8G6	mAb-2H10	mAb-10D12	LY-CoV555	REGN10987	REGN10933
WT（IC_{50}μg/ml）	0.10	0.08	0.29	0.08	0.09	0.33	0.07	7.18	0.04	0.01	0.03	0.03	0.02
Alpha（IC_{50}μg/ml）	0.87	0.27	0.31	0.05	0.07	0.10	0.03	3.60	0.09	0.27	0.03	0.01	0.01
Beta （IC_{50}μg/ml）	1.08	>10	0.55	0.15	0.15	>10	0.05	>10	4.14	>10	>3.33	0.01	0.57
Gamma（IC_{50}μg/ml）	0.15	>10	0.32	0.03	0.04	>10	0.01	>10	3.36	>10	>3.33	0.02	0.96
Delta（IC_{50}μg/ml）	0.06	0.03	0.08	9.27	0.03	3.69	0.03	9.77	0.02	0.01	>3.33	0.04	0.01
Lambda（IC_{50}μg/ml）	0.03	0.05	0.12	0.05	0.05	7.15	0.03	4.34	0.02	0.01	>3.33	0.08	0.02
Mu（IC_{50}μg/ml）	0.45	>10	0.20	0.07	0.09	>10	0.03	>10	3.11	>10	>3.33	0.02	0.47
Omicron（IC_{50}μg/ml）	>10	>10	>10	7.77	9.47	>10	0.02	4.25	>10	>10	>3.33	>3.33	>3.33

Table S3. Neutralizing titers of the mAbs in the authentic virus neutralization assay

	mAb-10F9	mAb-1F9	mAb-2F7	mAb-7B8	mAb-4E5	mAb-9G11	mAb-9A8	mAb-8G6	mAb-2H10	mAb-10D12
WT（IC_{50}μg/ml）	0.16	0.06	0.24	0.08	0.12	0.24	0.16	5.21	0.08	0.06
Beta （IC_{50}μg/ml）	3.91	>125	1.30	0.16	0.49	>125	0.12	>125	>125	>125
Delta（IC_{50}μg/ml）	0.49	0.24	0.12	>125	0.49	20.83	0.33	>125	0.12	0.08
Omicron（IC_{50}μg/ml）	>125	>125	>125	>125	>125	>125	0.49	>125	>125	>125

Table S4. Statistics for cryo-EM data collection, refinement, and validation

	Omicron S trimer in complex with 9A8 (State 1)	Omicron S trimer in complex with 9A8 (State2)	9A8-RBD-interface
**Data collection and processing**			
Magnification	22,500	22,500	22,500
Voltage (kV)	300	300	300
Electron exposure (e–/Å^2)	60	60	60
Defocus range (μm)	-1.5--2.7	-1.5--2.7	-1.5--2.7
Pixel size (Å)	1.07	1.07	1.07
Symmetry imposed	C1	C1	C1
Initial particle images (no.)	2,815,543	2,840,573	347,106
Final particles images (no.)	494,849	339,195	380,727
Map resolution (Å)	3.4	3.6	4.3
FSC threshold	0.143	0.143	0.143
Map resolution range (Å)	3.4-60	3.6-60	4.3-60
**Refinement**			
Initial model used (PDB code)	7WEA	7WEA	7WEA
Model resolution (Å)	3.3	3.3	3.3
FSC threshold	0.143	0.143	0.143
Model resolution range (Å)	3.3-60	3.3-60	3.3-60
Map sharpening *B* factor (Å^2)	-148.4	-139.3	-239.0
Model composition			
Non-hydrogen atoms	28,339	30,264	3,359
Protein residues	3,523	3,752	430
Ligands	49	63	0
B factors (Å^2)			
Protein	82.72	89.12	110.64
Ligand	117.90	113.03	-
R.m.s. deviations			
Bond lengths (Å)	0.003	0.004	0.005
Bond angles (°)	0.671	0.762	1.264
Validation			
MolProbity score	1.93	1.79	1.79
Clashscore	9.07	6.01	5.46
Poor rotamers (%)	0.00	0.03	0.00
Ramachandran plot			
Favored (%)	93.02	92.73	91.75
Allowed (%)	6.89	7.19	8.25
Disallowed (%)	0.09	0.08	0.00

Table S5. List of interacting residues between 9A8 and Omicron SARS-CoV-2 RBD trimer (d < 4 Å)

Complex	Omicron RBD	Heavy chain		Light chain
**Omicron S-trimer in complex with 9A8**	T415T	S56	Y58	
	Y421Y	Y33	S53	
	L455L	Y33	G101	
	F456F	Y33		
	Y489Y	M106		
	Q493R	S102	S104	
	Y505H			N30
